# Bridging the gap between models and reality: development of a research environment for an object-oriented hospital information system to integrate artificial intelligence and robotics into clinical practice

**DOI:** 10.1007/s11548-025-03470-6

**Published:** 2025-07-03

**Authors:** Sidra Rashid, Lukas Bernhard, Sonja Stabenow, Emily Spicker, Charlotte Haid, Carl König, Hedi Louise Kramer, Sandro Pischinger, Daniel Schade, Johannes Fottner, Dirk Wilhelm, Maximilian Berlet

**Affiliations:** 1 MITI Research Group, TUM School of Medicine and Health, University Hospital rechts der Isar, Ismaninger Str. 22, 81675 Munich, Bavaria Germany; 2 Department of Surgery, TUM School of Medicine and Health, University Hospital rechts der Isar, Ismaninger Str. 22, 81675 Munich, Bavaria Germany; 3 Chair of Materials Handling, Material Flow, and Logistics, Technical University of Munich, Boltzmannstraße 15, 85748 Garching, Bavaria Germany; 4 TUM School of Computation, Information and Technology, Technical University of Munich, Boltzmannstraße 3, 85748 Garching bei München, Germany

**Keywords:** Model-guided medicine, Research framework, Hospital information system, Object-oriented representation, Pragmatic digital twin, Artificial intelligence, Medical robotics

## Abstract

**Introduction:**

Hospital information systems (HISs) are the main access opportunity for medical professionals to computer-based patient administration. However, current HISs are primarily designed to function as office applications rather than as comprehensive management and supporting tools. Due to their inflexible architecture, integrating modern technologies like artificial intelligence (AI) models and medical robotics (MR) is hindered. Therefore, we have conceptualized an object-oriented HIS (oHIS) as a pragmatic digital twin (PDT) of the entire patient care in a hospital and developed a functional research framework software for further investigations to bridge the gap between reality and models via oHIS.

**Material and methods:**

In an interdisciplinary team of engineers and physicians, we conducted a requirements assessment on the surgical wards of the TUM University Hospital in Munich. Then, we designed the research framework named *OMNI-SYS* and developed it into a functional research platform capable of bridging the gap between a model management system and real-world agents. Finally, we evaluated the framework simulating a clinical use case.

**Results:**

Our analysis revealed that future-proof HIS is an under-researched topic. The integration of new technologies into clinical practice is not sufficiently prepared. Therefore, our approach could solve this shortcoming allowing for human agents, devices, models, and robots to interact in a PDT. Models can be integrated as quasi-natural objects and interact with representations of tangible objects in real time. This approach enables even the integration of new technologies that are still unimaginable today. Our oHIS research framework enabled a functional object representation in a simulated use case.

**Conclusion:**

oHIS could significantly facilitate the integration of future technologies like AI models and MR. The OMNI-SYS framework could serve as a cornerstone for further research into this new approach. Studies on its clinical application and formalization are already planned in preparation for a possible future standard.

## Introduction

The use of digital information technology in managing patient data is a milestone in the history of medicine. Today, using electronic health records (EHR) is the standard rather than an exception in industrialized countries [1]. Hospital information systems (HISs) are software frameworks that manage EHR and make it available to medical professionals. Hence, HISs are the central access point to digitized inpatient healthcare [2].

Current HISs are mainly inspired by traditional hard copy patient records, as most programs historically originate from transferring printed and filed information to EHR administration systems (digital transformation) [3]. Current problems in implementing new technologies and extracting data for research result from this imposed legacy of the paper sheet [4, 5].

Despite numerous innovative achievements in artificial intelligence (AI) and medical robotics (MR), only a few reach the level of clinical application, as integration into existing HIS infrastructure and continuous further development pose significant challenges [6–11]. Interfaces to newly developed applications are often improvised and unstable, even for classic non-AI and non-MR services [12, 13]. Recent developments such as HL7 (FHIR) address the interoperability problem but do not examine the fundamental architecture and functionality of a future-proof HIS [14].

In 2006, Haux formulated that future HIS should integrate patients as active users, be compatible inter-institutionally, be usable for therapy planning, and shift the storage strategy from simple alphanumeric to molecular data formats. This culminates in the demand for international strategies for new HIS architectures, a patient-centric design, and forced research in biomedical informatics [15].

An example of a broader approach regarding the integration of new technologies into clinical practice is Healthcare 4.0 (H4.0), facilitating the shift from a hospital-centric to a patient-centric view of clinical data management [16]. A related approach concerning the implementation of AI in the healthcare sector refers to models and model-guided medicine. Knowledge-based models of patients, medical procedures, and even molecular processes are, primarily AI-driven, created to correspond as closely as possible to reality (truth) achieving as accurate as possible predictions concerning health-related problems. Lemke et al. highlighted the need for a structured and comprehensive Medical Information and Model Management System (MIMMS) in 2014 [17, 18]. However, this requires a continuous flow of data and information from reality to the virtual models and vice versa, which current HISs do not provide.

**Fig. 1:**
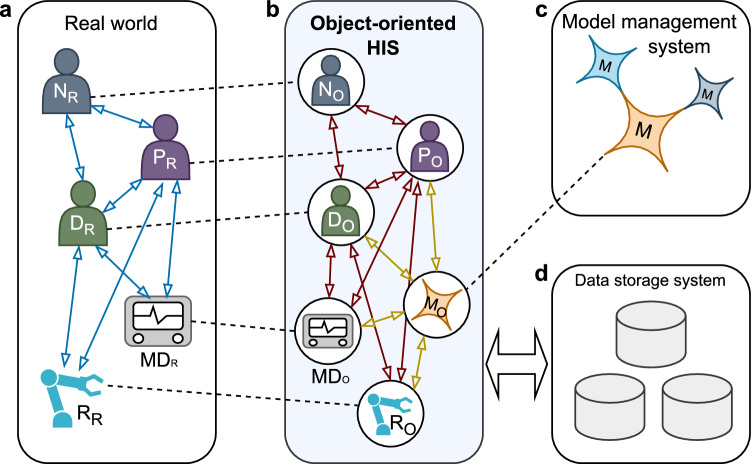
Concept of an object-oriented HIS that links models with the real world: a: doctors (D_R_), nurses (N_R_), patients (P_R_), medical devices (MD_R_), and medical robots (R_R_); b: object representations (X_O_); c: models (M); d: data storage system

A promising approach seems the use of a *pragmatic digital twin* (PDT) of all relevant human agents in a hospital environment, allowing for mirroring interactions of the various roles (doctors, nurses, and patients) and reflecting the results of these interactions continuously [19]. Following this principle, medical devices and new applications such as AI models and MR could similarly be integrated in the form of a virtual image (object representation) and interact with the virtual human representations. (Fig. 1)

The principle of object representation in computer science is proven and has a long tradition. Object-oriented programming (OOP) was implemented more than 40 years ago [20]. Here, classes and their instances representing abstract concepts or real objects are equipped with functions (methods) and data (properties) on the code level, allowing for interaction at program runtime. Applying these principles to the application layer of a new generation of HIS could be the missing link between models and reality, leading to simplified integration of future emerging technologies.

We have developed the research framework *OMNI-SYS* to examine fundamental principles, implications, and issues for the creation of future object-oriented hospital information systems (oHISs) within the scope of the *TUM4HealthTech* project granted by the Bavarian State Ministry of Science (grant number 1200/2022). This article reports on the conception, design, evaluation, and first conclusions regarding ideal oHIS architecture for bridging clinical practice and future technologies.

## Material and methods

### Composition of the development team

Throughout the entire developing process of the oHIS research framework, we followed the principle of *Surgineering*. The prime directive was interdisciplinarity that required a team of engineers and physicians making all design and development decisions. [21] Our team comprised five computer scientists, two medical engineers, two logistics scientists, and three physicians.

### Analysis of requirements

**Fig. 2:**
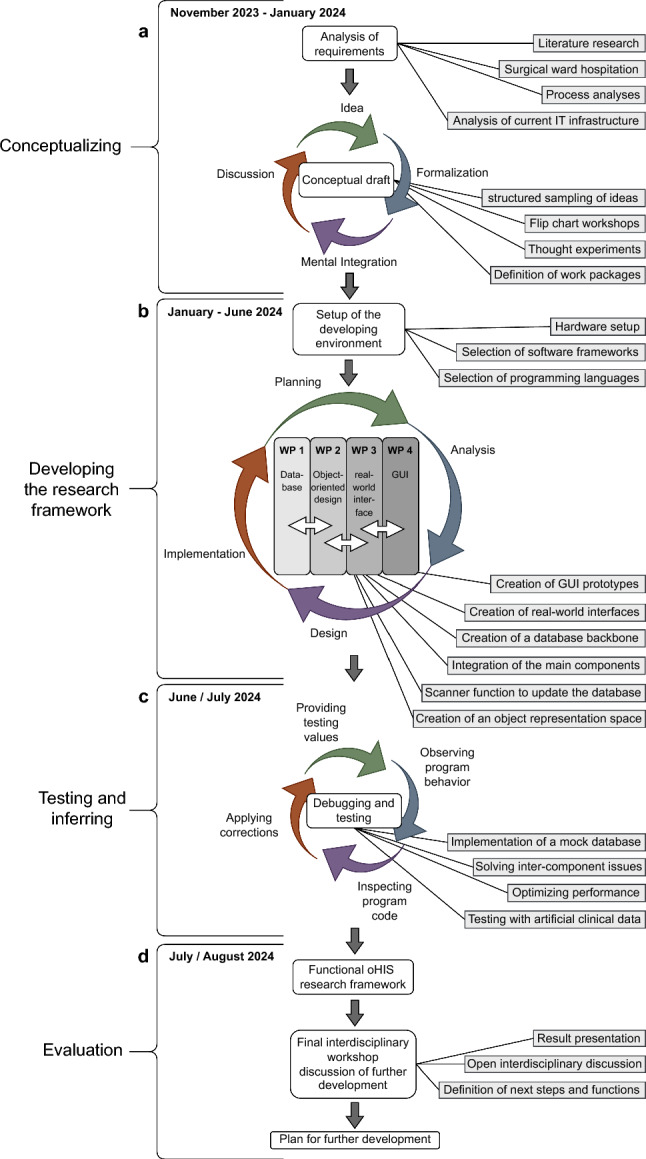
Development process leading to a functional research framework for advancing object-oriented hospital information systems (HISs)

The requirements analysis built upon three pillars: (1) We thoroughly reviewed the literature to explore the fundamental requirements for modern HIS, examining available market solutions and approaches for integrating emerging technologies. (2) An in-depth analysis of the processes in the surgical wards at the TUM University Hospital in Munich was conducted, aiming to achieve that essential processes were incorporated in the research framework. (3) Through interdisciplinary collaborative workshops, a foundational philosophy for the planned research framework was established, along with delineating the required basic functionalities (Fig. 2a).

### Conceptual draft

Conceptualizing the foundational design spanned three months and involved regular discussion workshops. Notably, the basic design intentionally excluded interviews with non-involved medical professionals to preserve its innovative essence, as the objective was to create an entirely new design rather than merely optimizing existing approaches (Fig. 2a).

### Planning the programming and distribution of tasks

To enhance efficient programming, we organized the development into four distinct work packages (WP) that were executed simultaneously. Acknowledging the interdependent nature of the functions, we conducted weekly collaborative workshops to ensure effective coordination among the WP that were designated as follows: (1) *Object-Oriented Design and Implementation of Core Modules of a Real-Time Agent-Based Model of a Dynamic Healthcare Environment*, (2) *Data Modeling and Database Design of a Real-Time Agent-Based Model of a Dynamic Healthcare Environment*, (3) *Exploring Real-Life Data Integration for Real-Time Agent-Based Modeling of Healthcare Environments*, and (4) *User Interface Prototype Development and Usability Testing*.

### Developing environment and technologies

The oHIS research framework was developed as a web-based application to enable easy access from any computer or handheld device. *Python* and the framework *Quart* were chosen due to their rapid and asynchronous prototyping capabilities and support for OOP [22]. To store virtual representations of objects, their properties, and their interactions, we developed a simulated graph database using the *Neo4j* toolkit, modeling objects within specific contexts, locations, and time frames. To enable real-time simulation of activity changes, QR code scanners were integrated as a link between real objects and their virtual representations. The Neo4j toolkit was used to create a graph database, a NoSQL-based approach optimized for storing connected data as nodes (entities) and edges (relationships). Graph databases are uniquely different from relational databases in terms of their ability to store relationships and connections as first class entities. Since Neo4j is the most widely adapted graph database approach in industry, we chose it for simulating the interactions between the objects. However, graph databases face significant challenges in scalability. As the dataset grows, distributing data across multiple machines becomes increasingly complex due to the interconnected nature of nodes and edges. Moreover, while the flexibility of supporting various graph types is an advantage, it can also complicate the data representation and processing. The technology is still maturing in areas such as performance optimization and distributed computing, especially when compared to relational databases [23]. Despite these limitations, Neo4j was well-suited for our research into object-related interactions (Fig. 2b)

### Testing and debugging

Throughout the software development process, a continuous integration (CI) and continuous deployment (CD) pipeline was established using GitLab CI/CD to automate the build and testing workflows. For testing, *Pytest* was used, particularly to verify the endpoints of the application and handle the asynchronous test cases effectively. Alongside debugging and testing, the software was reviewed for its functionality regarding usability by medical professionals during weekly team workshops (Fig. 2c).

### Evaluation of the results

Once the final framework was completed, we conducted simulations of various use cases inferred in the Neo4j graph database with object representations of patients, doctors, nurses, robots, and models. Thus, we aimed to simulate future object interactions like examinations or granting access to patient data for AI models and robots. The results were presented and discussed during a concluding interdisciplinary workshop. The involved physicians were interviewed using structured questions after testing the prototype for their perception, understanding of the underlying principles, and opinion about the potential of the object-oriented PDT for clinical routine. Drawing on the current state of the research framework, we identified a broad potential to further develop the concept of an object-oriented HIS and explored opportunities for future applications (Fig. 2d).

**Table 1:** Basic requirements for an object-oriented hospital information system

Topic	Demand	Field of tension
Usability and Safety	Ease of data access	Data security $\Longleftrightarrow$ Accessibility of data
	Ease of use	Complex processes $\Longleftrightarrow$ Intuitive design
	Data privacy	Data availability $\Longleftrightarrow$ Data autonomy
Expandability and Scalability	Easy function implementation	Stability $\Longleftrightarrow$ Updating during runtime
	Flexible model integration	Complex data $\Longleftrightarrow$ Access for models
	Flexible MR integration	Complex environment $\Longleftrightarrow$ Integration of MR
Architecture and data structure	Inclusion of all stakeholders	Assignment of responsibilities $\Longleftrightarrow$ Flexibility
	Pragmatic object representation	Object definition $\Longleftrightarrow$ Complex environment
	Real-time functionality	In-time reaction $\Longleftrightarrow$ Simultaneous tasks

## Results

### Requirements for an object-oriented HIS

Following Haux [15], the expansion into regional and global health information systems, the integration of patients as active users, and the enhancement of functionality beyond basic administration to encompass proactive therapy planning are not yet fully addressed by current HIS solutions. Particularly relevant are the demands for strategic information management, the expansion of data modalities to the molecular level, and a strategy for including new, even sensor-based technologies.

Regarding the acceptance of HIS, human factors have a more pronounced impact than organizational or financial factors. A fundamental understanding of how IT solutions improve work processes is crucial. From a technical point of view, enabling continuous development focused on future functionalities and the reliability of data management are the most significant elements regarding acceptance [24].

Clinical software solutions that facilitate the seamless replacement of outdated applications with advanced, e.g., AI-driven solutions would be highly advantageous, particularly in terms of ensuring their maintainability within clinical practice [25]. Particularly, the sustainable integration of medical robotic systems into clinical routine remains under-researched. Nonetheless, it is essential to develop systematic implementation strategies for MR, as these systems offer significant potential to enhance quality in patient care [26].

Process analyses on the surgical wards of the TUM University Hospital in Munich supplemented the results of the literature research. The expert team then formulated basic requirements for oHIS within several focus group discussions (Table 1). It became apparent that the most significant challenges lay in the tension between a complex hospital environment and the demand for the most straightforward and intuitive design.

### Fundamental principles

The fundamental principles of an oHIS must remain valid with the integration of future technologies that are still unimaginable today. We overcame this issue by applying the dimensions of the real world to the PDT. Dimensions like location and time will never change as they are imposed by natural laws. Thus, we introduced a *Location* dimension to represent the state that a particular object is situated in a defined *Location* at a specific *Time*.

**Fig. 3:**
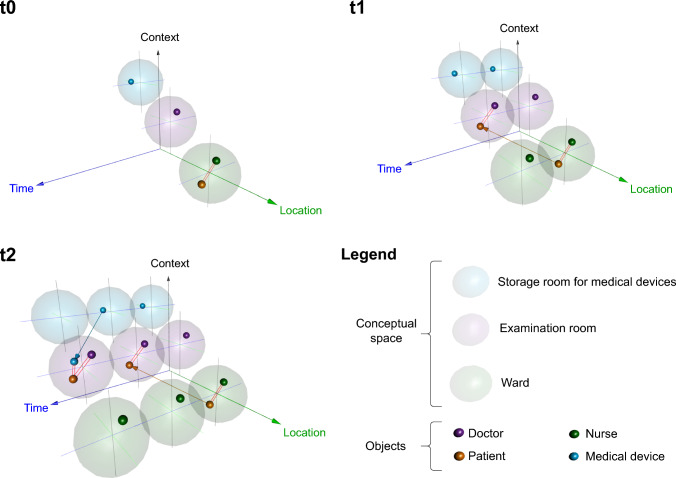
Concept of object representation and object interaction in the three-dimensional space of Time, Location, and Context: t0 (Timepoint 0): An ultrasound device (blue), a doctor (purple), and a patient (orange) are situated in the separated contextual and local spaces storage (transparent blue), examination room (transparent purple), and ward (transparent green). The patient is interacting (red lines) with a nurse (green). t1: The patient moves to the examination room and interacts with the doctor. t2: The ultrasound device moves from the storage to the examination room and interacts with the patient

However, to comprehensively map the processes in a hospital, a further dimension was required, namely that of ’purpose’ or ’meaning’. No human or material object exists without context, even when waiting for an examination or being stored for later use. Thus, the third dimension of the oHIS framework is that of *Context*. Hence, all objects operate at runtime in an abstract space that is spanned between *Time*, *Location*, and *Context*. Figure 3 illustrates interactions among objects within a virtual space.

The next step was implementing the representation of actually existing agents and objects. Here, we strictly followed the principles of OOP. The main ideas of OOP are *encapsulation*, *inheritance*, *information hiding*, *data abstraction*, and *polymorphism*. In order for an object to be created, it must belong to a class defined in the program. A class defines a set of methods and data that all objects created from it will contain. Therefore, encapsulation is the bundling of data and methods. It hides inner details and only exposes what is necessary, restricting direct access to some components, promoting modularity and preventing unintended interference.

**Fig. 4:**
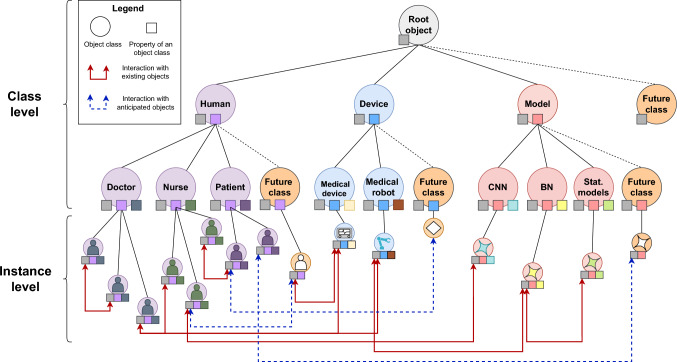
Hierarchical classification structure for current and future anticipated object representations in an object-oriented hospital information system (HIS)

Classes can inherit methods and data from other classes. For example, if the *Doctor* class inherits from a *Person* class, then the *Doctor* class will also have all the characteristics of the *Person* class. Hence, the ultimate superclass provides the most abstract interpretation of what it means for an object to be of a certain category. Taking this concept of abstraction from OOP principles, we model the agents flexibly by starting from an abstract root class from which many different classes can be derived. Accordingly, we included a *future object* class to anticipate technological applications or roles within patient care that do not yet exist. Thus, future objects and models can be registered when available without compromising the system’s stability. (Fig. 4)

One of the key concepts in OOP is *polymorphism*, which allows objects to take on different forms, enabling the same method or interface to work differently, depending on the object it interacts with. For example, a *Doctor* class with a method like *Examine Patient* could have subclasses such as *Pediatrician* or *Surgeon*, each providing a specialized implementation of *Examine Patient*.

Another OOP principle, *information hiding*, restricts access to internal object details through a controlled interface. For instance, a *Patient* class containing sensitive data like medical history might provide summary methods instead of direct access, ensuring data protection while maintaining simplicity and security. This approach promotes modularity and robustness [27].

Thus, inspired by OOP, we propose modeling the hospital as a system of interconnected components represented as objects with attributes and functionalities that interact within specific contexts and timepoints.

### Functionality of the prototype

Following the work packages, the OMNI-SYS research framework includes a virtual space spanned by *Time*, *Location*, and *Context* and a comprehensive object representation for people, things, and models. In the first functional version, the person groups of patients, nurses, and doctors are already considered central actors. On the ’things’ and ’model’ side, non-human objects like robots and models of all kinds can be registered in the system. Object interactions can be transferred from the real world to the conceptual space by updating the system’s status as objects in the real world move to the same location and interact in the same context. This is made possible using QR codes or RFID tags, meaning that no input via a GUI is initially required during an interaction in the real world. The natural interaction is disturbed as little as possible.

**Fig. 5:**
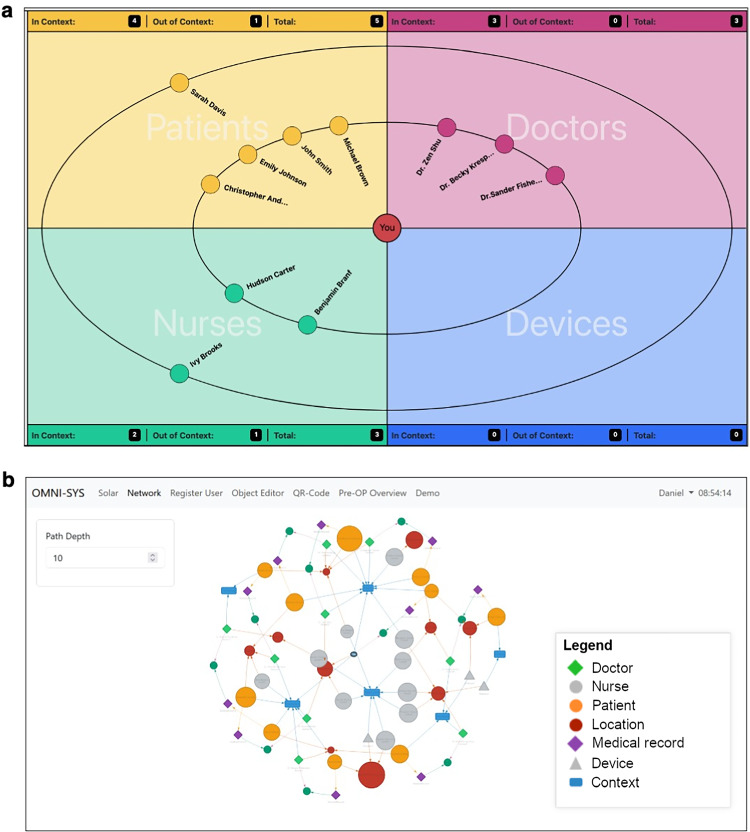
Two exemplary GUI prototypes of the oHIS research framework. **a** Solar system view. **b** Network view

To document examinations and create orders, as well as to provide an overview of all objects (devices and persons) in their own area of responsibility for medical professionals, we have created an exemplary intuitive GUI prototype in which the user is always situated in the center. Other relevant objects are displayed in a view similar to the solar system (solar view) surrounding the user like planets. When the user hovers over an object, more details regarding its properties can be viewed. The GUI prototype is displayed in Fig. 5a.

In addition to the *solar system view*, Fig. 5b displays a more scientific *network view* that also depicts objects and their relationships relative to the central user. Moreover, in this view all detailed interactions, relations and properties of surrounding objects are displayed with a deliberate and variable path depth. In this network view, both the location and context are also displayed as nodes to reach a flat, non-3D rendering of the 3D conceptual space shown in Fig. 3. Objects such as doctors, nurses, devices, models, and patients can be related to more than one context to mirror reality as exactly as possible. It is, therefore, a projection of the 3D representation of objects in space, time, and context onto a graph database view, displaying objects moving in real time.

Already the current version of the OMNI-SYS framework provides a broad opportunity for testing the idea of oHIS by intuitively changing the properties and relationships of the displayed objects within the graph network. It also provided an insight into the way data is related to the particular objects. For example, in line with the principle of data encapsulation, examination data such as sonography results would be exclusively stored within the corresponding patient object to achieve data security and privacy. The network view allows one to examine which kind of data belongs to a particular object. In addition, several GUI applications can be easily adapted within the research framework requiring small programming effort.

Finally, we designed an interface to edit object properties and relationships for quick testing and evaluation of object-oriented oHIS concepts. This feature allows users to modify object attributes and instantly view the changes in the network and solar view, eliminating the need to write queries to the graph database.

### Integration of artificial intelligence models and medical robotics

**Fig. 6:**
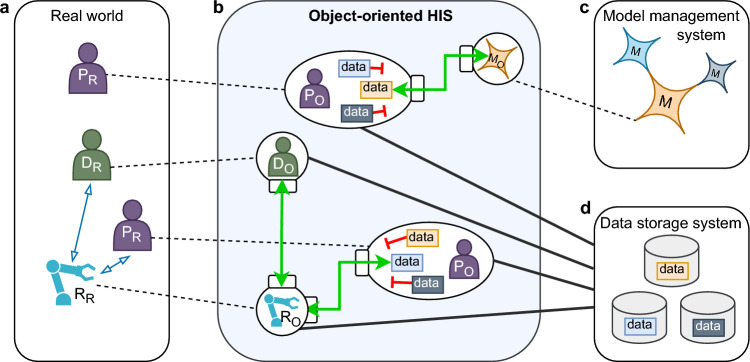
Integration of artificial intelligence models and medical robotics into the object-oriented HIS. **a** Interacting agents in the real world. **b** Object representations in the oHIS. **c** Integrated AI models. **d** Data storage

A fundamental goal of developing an oHIS is the simple and future-proof integration of AI models and MR in a clinical environment. In principle, these two key technologies are integrated the same way as other existing agents. Every robotic system and every model also receive an object representation in the oHIS that is compatible with all other objects, allowing to interact as if they were real existing agents themselves. In future, robotic systems could access relevant patient and user data to adapt their behavior in the real world. If a robot or an AI model is defective, it can be removed by deactivating the object representation at runtime without affecting the integrity of the overall system. (Figs. 4 and 6)

**Fig. 7:**
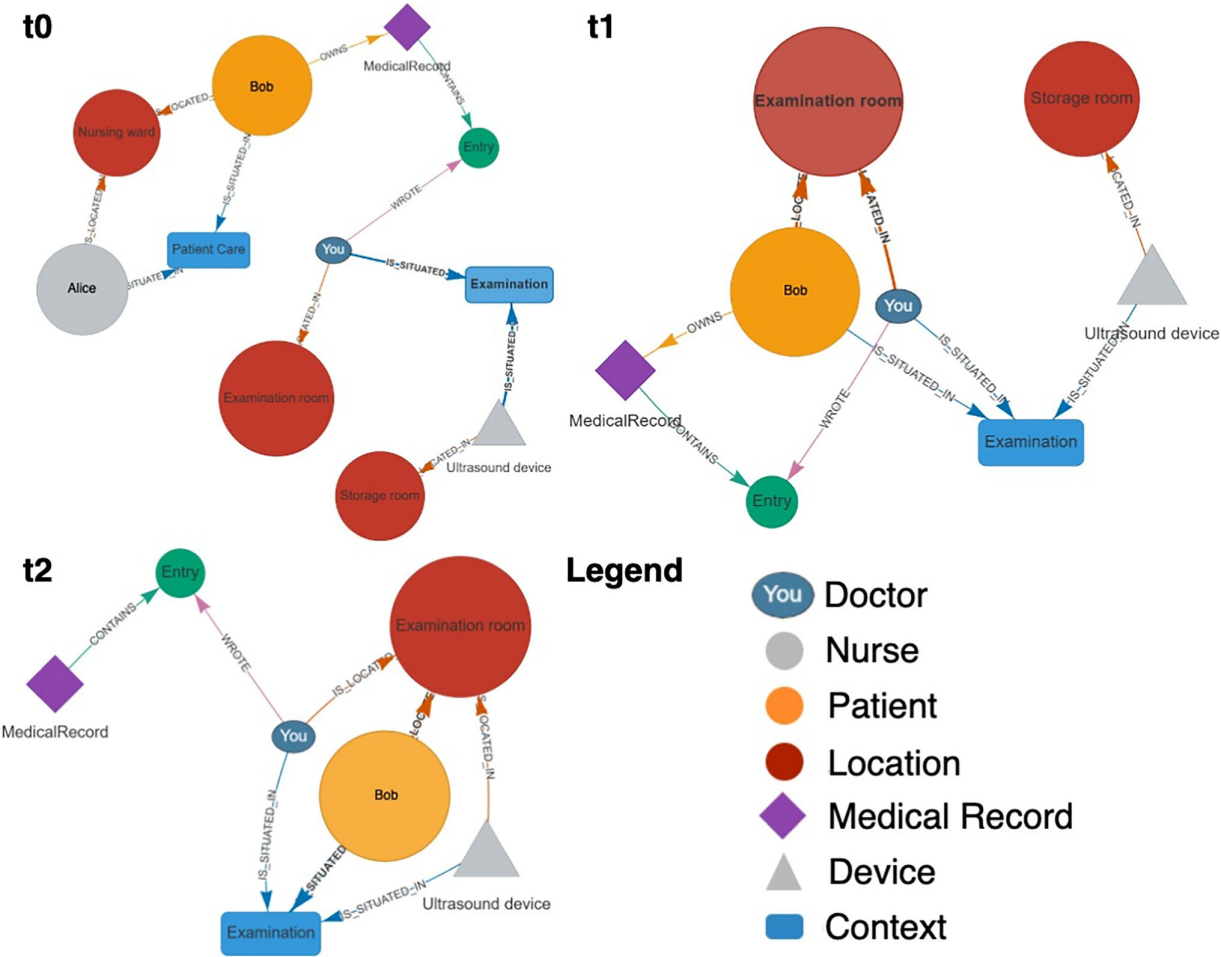
Object representation and interaction in the 3D space of *Time*, *Location*, and *Context*: t0 (Timepoint 0): Main user (blue dot) in the examination room and examination context, patient (yellow dot) and nurse (gray dot) in the nursing ward and patient care context. Ultrasound device (gray triangle) in the storage but linked to examination context. t1: Patient moves to the examination room, interacts with the doctor. t2: Ultrasound device moves to the examination room and interacts with the patient

### Evaluation of the prototype

The prototype was evaluated using the sonographic examination use case shown in Fig. 3 with results displayed in Fig. 7. Objects such as doctor, nurse, ultrasound device, and patient were simulated as nodes in the graph database, along with *location* and *context*. Relationships between objects were represented as directed edges. For example, at time t0, the patient ‘Bob’ is linked to the location ‘Nursing ward’ via the edge located_in and to the context ‘Patient care’ via situated_in. Other relationships were also simulated, such as ‘Bob’ owning a medical record written by the doctor. The prototype was tested by team members acting as ‘Bob’ (the patient) and the doctor. QR codes were created for each location and context, and for the patient, encoding their unique ID.

During evaluation, the actor patients, recruited from our team, used their phone’s camera to scan a QR code containing a link that sent an HTTP request to the web-based application, registering them in the system. Initially located in the nursing ward, the patient’s goal was to move to the examination room for an ultrasound. Upon arrival, the patient scanned the examination room’s QR code to update their location and context. Since the patient had already scanned their identification QR code, the system used cached cookies to recognize them and update their location accordingly.This change shown in Fig. 7 removed the nursing ward and the nurse nodes from patient object since they were no longer relevant to the main user (doctor). Also the ultrasound device was registered as object. The system reliably logged changes in object properties and relationships, along with timestamps, enabling the collection of a comprehensive event-based dataset. Such a dataset can easily be utilized for process mining techniques to model workflows within the hospital environment, offering valuable insights into potential areas for improvement. In future, e.g., sensors that measure vital signs could be integrated into the system, linking patient objects to the corresponding sensor data and AI models could instantly evaluate the device signals stored as properties of the related patient object. Although a phone-based QR scanner was used during prototype evaluation, it is not intended to be the sole method of data input. Further development will explore alternative interfaces such as sensors or patient wristbands with RFID chips to support patient registration and data capture. This is particularly important in cases where patients are not independent or are unable to interact with the system directly, such as those who are unconscious. Moreover, patient registration in this case was linked to the registration of the patient’s location. However, the medical data of the patient are still the responsibility of the doctor to be entered into the system.

The graph database approach was efficient to mirror the interactions and localizations of the objects involved properly regarding this use case. However, for real-world implementations, especially in critical domains where consistency, availability, scalability, and security are crucial [28], relational databases may offer a more robust solution [29]. Their vertical scalability helps avoiding the complexities that arise as interconnected nodes and edges increase in graph databases [29].

The evaluation results were finally presented and discussed in an interdisciplinary team meeting at the end of the project period to set the direction for further development of the research framework. The most important conclusions are addressed in the discussion section.

Structured interviews with the involved physicians indicated a high degree of acceptance and understanding of the principles of oHIS. The proposed GUIs were considered as already functional; however, extended functionality was demanded when aiming to integrate an oHIS into clinical routines.

## Discussion

In this article, we report the development of a research environment on object-oriented hospital information systems (oHISs) as a basis for the future integration of emerging technologies, such as medical robotics and artificial intelligence, into everyday clinical practice. In the following discussion, we will explore the potential of this innovative concept and give insights into advised further development.

Our literature research has shown that the topic of future-proof HIS that anticipate the rapid evolution of medical AI and robotics is under-researched and that current developments in the clinical software sector are mainly inspired by the translation from paper to computer-based data administration. A HIS is thus perceived more as an office application rather than a comprehensive medical management and supporting tool. This degradation is the main problem with integrating new technologies into clinical practice.

Because an object-oriented HIS would consider patients, doctors, and nurses as intrinsic entities, this approach goes far beyond mere data administration. It changes the entire concept of managing medical information by replacing classic paper-like and form-dependent output with flexible and just-in-time reporting derived from data as original as possible.

Data sets, e.g., on medical examinations, would be encapsulated in the object of the corresponding patient and supplemented by the comments of the responsible physician making the assessment. The printout of an electrocardiogram (ECG) will no longer be stored as a document. Instead, the derived signal in the form of vector data would become the property of the specific patient object, fulfilling Haux’s demand for a smaller-scale data representation ready for proper tokenization regarding AI-based processing [15, 30].

The question for the best database format for oHIS cannot be fully answered yet. While graph databases offer structural flexibility and are well-suited for modeling dynamic, interconnected entities, relational databases remain the industry standard due to their compatibility with various programming languages. This compatibility is crucial for integrating diverse software applications, AI components, medical robotics, and devices that may be developed using different technologies. Ultimately, the choice of a database system for managing dynamic object states is a topic that warrants further investigation. Our framework is specifically designed to support this exploration, enabling comprehensive testing and evaluation across use cases. Future work will include systematic assessment of relational database systems in terms of scalability, latency, and security.

The data sovereignty at runtime would lie solely with the human agent being mirrored in the system. This allows patients to control their data autonomously and, for example, approve or reject access by statistical or artificially intelligent models and human agents in the context of studies or their treatment process.

Regarding responsible data handling, the concept of a *pragmatic* digital twin (PDT) is crucial to emphasize. This approach involves tracking only pertinent information about the depicted object, such as changes in location, context, or the addition of new data, rather than monitoring every aspect and movement continuously. The prototype of the research framework already provides this functionality through scanning QR codes or registering RFID chips, thereby minimizing both computational demand and data traffic associated with object updates. Additionally, the PDT addresses significant data privacy concerns: Employees and patients are not constantly tracked. Their restroom visits or location during breaks cannot be monitored. *Location* and *Context* updates occur only when changes relevant to the workflow arise.

When designing a future-proof oHIS, current AI and MR developments should not be perceived as the culmination of technological evolution. Technologies that seemed unimaginable 50 years ago have now been integrated into clinical practice (e.g., sonography and computed tomography) Considering the unpredictable further development of medical technology and informatics advancements during the next 50 years, it is essential to incorporate this uncertainty into conceptualizing future HIS. The concept of hierarchical classes, which can be expanded at any time, is well-suited for this purpose. Creating an abstract *future object* class containing communication interfaces with existing instances is already possible in our first OMNI-SYS version. This class can be enhanced with additional functions and properties, facilitating a comprehensive object representation as development progresses and allows for an assessment of the potential impact of integrating the particular technology into the PDT during runtime without compromising the integrity of the overall system.

The most important finding seems to be the paradigm that *everything* and *everybody* relevant for clinical workflows should be integrated in future hospital information systems using object representation. Thus, transferring OOP principles to the architecture of future oHIS solutions could lead to a flexible and extensible digital healthcare environment. Our research framework can serve as a tool to examine fundamental principles before developing productive software applications for daily clinical use. However, not only these apparent results of our project should be emphasized. The way and the strategy in which the prototype of the framework and the recommended architecture of an oHIS were developed are also meaningful. The interdisciplinary assessment of concepts and components with physicians actually working in clinical practice harbors the great opportunity of future translation into clinical practice, as clinical expediency is always ensured alongside technical innovation. In the case of developing productive object-oriented HIS, the strategy of longitudinally interdisciplinary development must be continued [31].

To further develop the approach of oHIS, further interdisciplinary collaboration on a national and international level is necessary to thoroughly consider all aspects of inpatient healthcare. The oHIS concept could even be expanded to a more comprehensive approach, going far beyond the frontiers of hospitals and expanding to general healthcare. Thus, even an object-based national or international representation of healthcare could become possible, bridging the gap between technology, models, and human agents.

## Conclusion

Our research framework OMNI-SYS demonstrates the feasibility of a novel object-oriented HIS approach in a simulated use case, highlighting its potential to address key shortcomings of current HIS architecture. This framework lays the foundation for adaptable and modular systems with future research focusing on clinical applications and formalizing object-orientation as a potential standard for HIS.

## Data Availability

The source code of the research framework is available on request from the authors.
